# Supplementary material for: Mitigation Actions Scenarios Applied to the Dairy Farm Management Systems
Source: Foods. 2023 Apr 29;12(9):1860. doi: 10.3390/foods12091860 (PMC10178217; doi:10.3390/foods12091860)
Supplement: Supplementary file 1 [file foods-12-01860-s001.zip › foods-2362042-supplementary.pdf]

Supplementary Materials

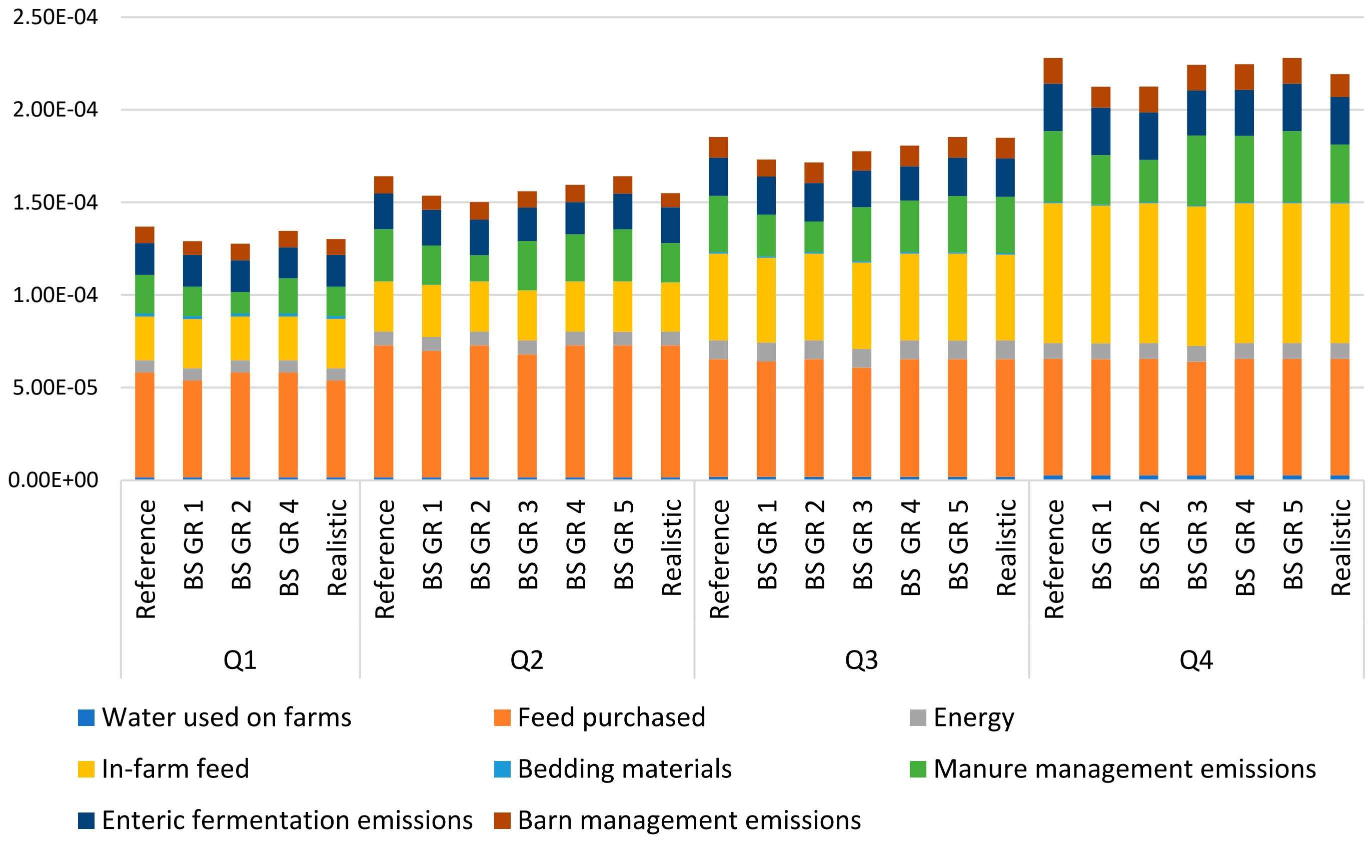

Figure S1. Potential reduction of the selected farm process by applying the five mitigation actions for the different Scenarios in each quartile Q1, Q2, Q3, Q4. Abbreviations: Reference: Reference Scenario; BS GR 1: Best-Case Scenario applying group 1; BS GR 2: Best-Case Scenario applying group 2; BS GR 3: Best-Case Scenario applying group 3; BS GR 4: Best-Case Scenario applying group 4; BS GR 5: Best-Case Scenario applying group 5; Realistic: Realistic Scenario.

Table S1. Weighted results per 1 of FPCM referred to the potential reduction of impact by applying the five mitigation actions for the different Scenarios in each quartile Q1, Q2, Q3, and Q4.

		CC	OD	IR, HH	POF	PM	A	FE	ME	LU	WRD	M-RD	F-RD	Total
Q1	Reference	4,15E-05	3,00E-09	1,79E-07	9,68E-06	8,81E-06	7,08E-06	1,38E-06	6,29E-06	1,53E-05	9,02E-06	3,27E-05	4,61E-06	3,49E-07
	BS GR 1	4,09E-05	2,78E-09	1,66E-07	8,10E-06	8,38E-06	6,29E-06	1,32E-06	5,92E-06	1,02E-05	8,53E-06	3,45E-05	4,35E-06	3,30E-07
	BS GR 2	3,24E-05	3,00E-09	1,79E-07	9,58E-06	8,81E-06	7,08E-06	1,38E-06	6,29E-06	1,53E-05	9,02E-06	3,27E-05	4,61E-06	3,49E-07
	BS GR 4	3,92E-05	3,00E-09	1,79E-07	9,65E-06	8,81E-06	7,08E-06	1,38E-06	6,29E-06	1,53E-05	9,02E-06	3,27E-05	4,61E-06	3,49E-07
	Realistic	4,09E-05	2,78E-09	1,66E-07	9,25E-06	8,38E-06	6,29E-06	1,32E-06	5,92E-06	1,02E-05	8,53E-06	3,45E-05	4,35E-06	3,30E-07
Q2	Reference	6,20E-05	2,02E-09	2,12E-07	1,00E-05	9,66E-06	7,22E-06	1,46E-06	6,77E-06	1,76E-05	1,18E-05	3,16E-05	5,39E-06	4,13E-07
	BS GR 1	6,14E-05	1,98E-09	2,03E-07	8,20E-06	9,32E-06	6,35E-06	1,42E-06	6,48E-06	1,06E-05	1,15E-05	3,25E-05	5,20E-06	3,79E-07
	BS GR 2	4,81E-05	2,02E-09	2,12E-07	9,83E-06	9,66E-06	7,22E-06	1,46E-06	6,77E-06	1,76E-05	1,18E-05	3,16E-05	5,39E-06	4,13E-07
	BS GR 3	5,91E-05	1,95E-09	2,02E-07	9,49E-06	9,10E-06	6,72E-06	1,38E-06	6,26E-06	1,63E-05	1,12E-05	3,07E-05	5,11E-06	3,99E-07
	BS GR 4	5,73E-05	2,02E-09	2,12E-07	9,94E-06	9,66E-06	7,22E-06	1,46E-06	6,77E-06	1,76E-05	1,18E-05	3,16E-05	5,39E-06	4,13E-07
	BS GR 5	6,19E-05	2,02E-09	2,12E-07	9,99E-06	9,66E-06	7,22E-06	1,46E-06	6,77E-06	1,76E-05	1,18E-05	3,16E-05	5,35E-06	4,13E-07
	Realistic	6,18E-05	2,02E-09	2,12E-07	8,26E-06	9,65E-06	6,70E-06	1,46E-06	6,68E-06	1,09E-05	1,18E-05	3,16E-05	5,37E-06	4,08E-07
Q3	Reference	5,69E-05	2,02E-09	2,09E-07	1,16E-05	1,07E-05	7,67E-06	1,67E-06	7,38E-06	2,05E-05	1,17E-05	5,11E-05	5,40E-06	4,06E-07
	BS GR 1	5,67E-05	1,94E-09	2,07E-07	9,61E-06	1,04E-05	6,83E-06	1,66E-06	7,24E-06	1,24E-05	1,14E-05	5,11E-05	5,33E-06	3,02E-07
	BS GR 2	4,33E-05	2,02E-09	2,09E-07	1,15E-05	1,07E-05	7,67E-06	1,67E-06	7,38E-06	2,05E-05	1,17E-05	5,11E-05	5,40E-06	4,06E-07
	BS GR 3	5,56E-05	1,91E-09	1,94E-07	1,10E-05	9,97E-06	7,06E-06	1,59E-06	6,98E-06	1,87E-05	1,13E-05	4,98E-05	5,11E-06	3,73E-07
	BS GR 4	5,24E-05	2,02E-09	2,09E-07	1,16E-05	1,07E-05	7,67E-06	1,67E-06	7,38E-06	2,05E-05	1,17E-05	5,11E-05	5,40E-06	4,06E-07
	BS GR 5	5,69E-05	2,02E-09	2,09E-07	1,16E-05	1,07E-05	7,67E-06	1,67E-06	7,38E-06	2,05E-05	1,17E-05	5,11E-05	5,37E-06	4,06E-07
	Realistic	5,69E-05	2,02E-09	2,09E-07	1,16E-05	1,07E-05	7,64E-06	1,67E-06	7,38E-06	2,01E-05	1,17E-05	5,11E-05	5,40E-06	4,06E-07
Q4	Reference	6,57E-05	2,33E-09	1,80E-07	1,35E-05	1,11E-05	7,91E-06	5,33E-06	7,28E-06	2,54E-05	1,91E-05	6,67E-05	5,11E-06	6,29E-07
	BS GR 1	6,57E-05	2,32E-09	1,80E-07	1,09E-05	1,11E-05	7,03E-06	5,33E-06	6,24E-06	1,45E-05	1,91E-05	6,67E-05	5,11E-06	6,18E-07
	BS GR 2	5,03E-05	2,33E-09	1,80E-07	1,33E-05	1,11E-05	7,91E-06	5,33E-06	7,28E-06	2,54E-05	1,91E-05	6,67E-05	5,11E-06	6,29E-07
	BS GR 3	6,36E-05	2,48E-09	1,82E-07	1,35E-05	1,10E-05	7,89E-06	5,28E-06	6,96E-06	2,54E-05	1,89E-05	6,59E-05	5,07E-06	6,58E-07
	BS GR 4	6,23E-05	2,33E-09	1,80E-07	1,35E-05	1,11E-05	7,91E-06	5,33E-06	7,28E-06	2,54E-05	1,91E-05	6,67E-05	5,11E-06	6,29E-07
	BS GR 5	6,57E-05	2,33E-09	1,80E-07	1,35E-05	1,11E-05	7,90E-06	5,33E-06	7,28E-06	2,54E-05	1,91E-05	6,67E-05	5,10E-06	6,29E-07
	Realistic	6,57E-05	2,33E-09	1,80E-07	1,19E-05	1,11E-05	7,39E-06	5,33E-06	7,28E-06	1,87E-05	1,91E-05	6,67E-05	5,11E-06	6,29E-07
